# Preparation of Ocimum Sanctum-Based Hydrogel and Evaluation of Its Cytotoxicity: An In Vitro Study

**DOI:** 10.7759/cureus.48110

**Published:** 2023-11-01

**Authors:** Devika Bajpai, Jaiganesh Ramamurthy

**Affiliations:** 1 Department of Periodontology, Saveetha Dental College and Hospitals, Saveetha Institute of Medical and Technical Sciences, Saveetha University, Chennai, IND

**Keywords:** herbal extract, periodontitis, local drug delivery, hydrogel, ocimum sanctum

## Abstract

Introduction: While traditional periodontal treatments like scaling and root planing, antibiotics, and surgical intervention remain the primary approaches, herbal medicine is continuously evolving as an alternative for the management of periodontal diseases. This study focused on the evaluation of the cytotoxicity of Ocimum sanctum (OS)-based hydrogel for its use in local drug delivery in periodontitis.

Methods: OS-based hydrogel was prepared using 50 g of OS powder and 200 ml of ethanol, along with carboxymethyl cellulose gel and sorbitol. The prepared formulation was evaluated for its cytotoxicity by using the MTT assay, cell viability, cell morphology, and brine shrimp egg assessment.

Results: Cell viability was assessed, and it was above 95% for the control and 85% for the OS hydrogel by the 3-[4, 5-dimethylthiazol-2-yl]-2, 5-diphenyltetrazolium bromide assay. Brine shrimp egg assessment also showed a survival rate of 80% at low concentrations. The cell morphology test showed round and uniform cells growing in a monolayer shape.

Conclusion: The findings of this study confirmed that OS-based hydrogel is cytocompatible and, hence, can be used as a local drug delivery agent for periodontitis management, especially in resource-constrained settings where affordable and natural treatment options are highly valued.

## Introduction

Periodontitis is a chronic inflammatory condition that affects the supporting structures of the teeth, including the gingiva, periodontal ligament, and alveolar bone. The condition is caused by the accumulation of dental plaque, a biofilm that forms on the teeth and contains various microorganisms [1]. The disease's development and progression are influenced by multiple factors, including the individual's immune response (host), the specific microorganisms present in the dental plaque, environmental factors like smoking, and genetic predisposition. Periodontal pathogens, along with other species, form a complex microbial community within dental plaque. The pathogens can initiate and sustain the inflammatory response that leads to the destruction of the periodontal tissues [2]. In order to treat periodontal disease, scaling and root planing are used to remove etiological elements such as plaque and calculus, thereby removing the biofilm consisting of microorganisms [3].

The use of systemic antibiotics is prescribed in the treatment of severe cases of periodontitis. The antimicrobials-Tetracyclines, Penicillins, Metronidazole, and Clindamycin-have been commonly used as single drug regimens in the early approaches to systemic antibiotics for periodontal therapy [4]. The decision to prescribe systemic antibiotics should be based on careful evaluation and proper diagnosis by a dental professional. While systemic antibiotics can be effective in controlling bacterial infections, there are potential risks associated with their use [5]. Insufficient retention of the active substance locally for long enough periods of time was one of the factors contributing to the poor outcomes of systemically administered drugs. If antimicrobial medicines could be applied locally, these downsides would be significantly reduced, while unfavorable side effects like gastrointestinal issues and the emergence of antibiotic resistance could also be reduced [6]. By including the active ingredient in controlled-release delivery devices that are inserted directly in the periodontal pocket, a drug's local tissue concentration can be increased. For locations thought to be challenging to instrument due to depth or anatomical complexity, such as furcation defects, sustained local delivery systems may also be suggested [7].

Herbal extracts have gained attention for their potential use as local drug delivery systems due to their natural bioactive compounds and therapeutic properties. Many researchers have already covered the use of herbal extracts for local medication delivery for periodontitis. According to Sastravaha et al., supplementary local administration of extracts from Purina granatum and Contella asiatica dramatically reduced the clinical symptoms of chronic periodontitis [8]. According to research by Kushiyama et al., drinking green tea has a negative correlation with periodontal disease [9]. After complete debridement, Yaghini et al. examined the use of an herbal gel based on Quercus brantii and Coriandrum sativum in periodontal pockets in individuals with moderate chronic periodontitis [10].

Indeed, Ocimum sanctum (OS), commonly known as Holy Basil or Tulsi, holds a prominent place in traditional medicine and is often referred to as the "Queen of Herbs" due to its numerous therapeutic properties. OS has a long history of use in various formulations to cure diseases and is described in ancient literature. The plant is considered sacred in many cultures and has been used for its medicinal benefits for centuries. It is native to the Indian subcontinent and is highly valued in Ayurveda, the traditional system of medicine in India. The herb has been the subject of numerous pharmacological trials, and research has supported its potential health benefits, such as anti-microbial, antioxidant, and anti-inflammatory effects [11].

The objectives of this study were to prepare and evaluate the cytotoxicity of OS-based hydrogel by means of different assays like the 3-[4,5-dimethylthiazol-2-yl]-2,5-diphenyl tetrazolium bromide (MTT) assay, the brine shrimp lethality assay (BLSA), and cell morphology. Therefore, this study aimed to prepare an OS-based hydrogel and evaluate its cytotoxicity.

## Materials and methods

Preparation of Ocimum sanctum hydrogel

Fifty grams of OS powder and 200 ml of ethanol were mixed and kept on the stirrer for 24 hours at room temperature. The supernatant was transferred into the beaker and kept under a water bath at 65°C for 30 minutes, and Ocimum sanctum extract was prepared. Carboxymethyl cellulose (CMC) gel was prepared by adding 2 gm of CMC powder in 100 ml of deionized (DI) water, which was mixed and kept under refrigeration for 24 hours. 0.5 gm of sorbitol and 0.5 gm of OS extract were added to 100 ml of CMC gel, which was mixed and kept in the refrigerator at 4°C for 24 hours. 90 ml of this mixture was poured onto the petri plates (three petri plates with 30 ml in each) and dried overnight at 37°C.

Cell lines

MG-63 cell lines were employed for the cytotoxicity test. MG-63 is a human osteosarcoma cell line commonly used in research studies related to bone biology and cancer. The MG-63 cell lines were cultured in a specific environment. The cells were allowed to grow exponentially in a humid environment with 7% carbon dioxide (CO2) and 93% air. The cells were grown in Roswell Park Memorial Institute (RPMI) 1640 medium with 10% fetal bovine serum (FBS).

MTT assay

**Figure 1:**
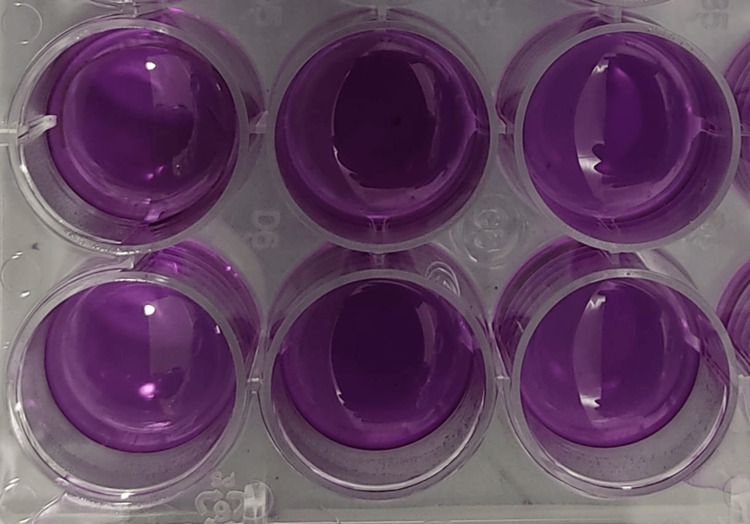
MTT assay The MG-63 cell line was used to perform a 3-[4, 5-dimethylthiazol-2-yl]-2, 5-diphenyltetrazolium bromide assay (MTT assay) for OS hydrogel cytotoxicity analysis.

The technique outlined by Mossman (1983) was modified. Trypsinization of the MG-63 cell line and repeated pipetting of the MG-63 cell line were used to acquire cell suspensions from exponentially expanding cultures of each cell line. In 96-well microtiter plates, cells were plated in 180-microliter media at the proper seeding density. The formazan product from the MTT substrate is produced by incubating each well for four days with 20 microliters of 10x OS gel in PBS (phosphate buffer solution), with PBS added to control wells. This process allows enough time for cell multiplication, OS-induced cell death, and loss of enzymatic activity (Mossman, 1983). Hundred micrograms of MTT solution were added to each well after three days of incubation, followed by 4 hours of incubation. The microliter plates were centrifuged at 450 rpm for 10 min, and the supernatant was discarded, leaving each well with 30 microliters of residual media. Each well received 150 microliters of 100% dimethyl sulfoxide (DMSO), which was used to re-solubilize MTT formazan crystals. After 5 minutes of plate shaking on a plate shaker, spectrophotometers were used to analyze the results (Figure 1).

Cell morphology

The cell lines (MG-63) used were maintained at a cell suspension of 105 g/mL in a medium containing 10% FBS. These cell lines were then treated with extract, and their morphological changes were observed under a phase contrast microscope.

Cytotoxicity test

Cytotoxic assessment experiment BSLA using brine shrimp (Artemia) eggs and the plant extract of OS (Holy Basil or Tulsi) was performed. The experiment aimed at testing the mortality rate of brine shrimp nauplii when exposed to different concentrations of the OS in situ gel. A fish tank was prepared with water, and iodide-free salt was introduced into a beaker to create the brine solution. This solution was used to hatch the brine shrimp eggs. Crystallized salt was added to the brine solution and stirred for 10 minutes until it was dissolved. A tiny amount of sodium bicarbonate (0.5 mg) as a buffer was added to the fish tank. Brine shrimp eggs were then added to the tank and allowed to hatch. The hatching process typically took around 24 hours, during which the eggs developed into nauplii. After 24 hours, the hatched brine shrimp nauplii were distinguished from any unhatched eggs. Ten nauplii were transferred to six-well macroplates. Different concentrations (10μl, 20μl, 30μl, 40μl, and 50μl) of the OS in situ gel were added to separate wells, while one well served as the control. The movement of the brine shrimp nauplii in each well was observed. Stable nauplii were then examined under a microscope to assess their micromovement, and the mortality rate of the brine shrimp nauplii in response to each concentration of OS in situ gel was calculated.

## Results

MTT assay

**Figure 2:**
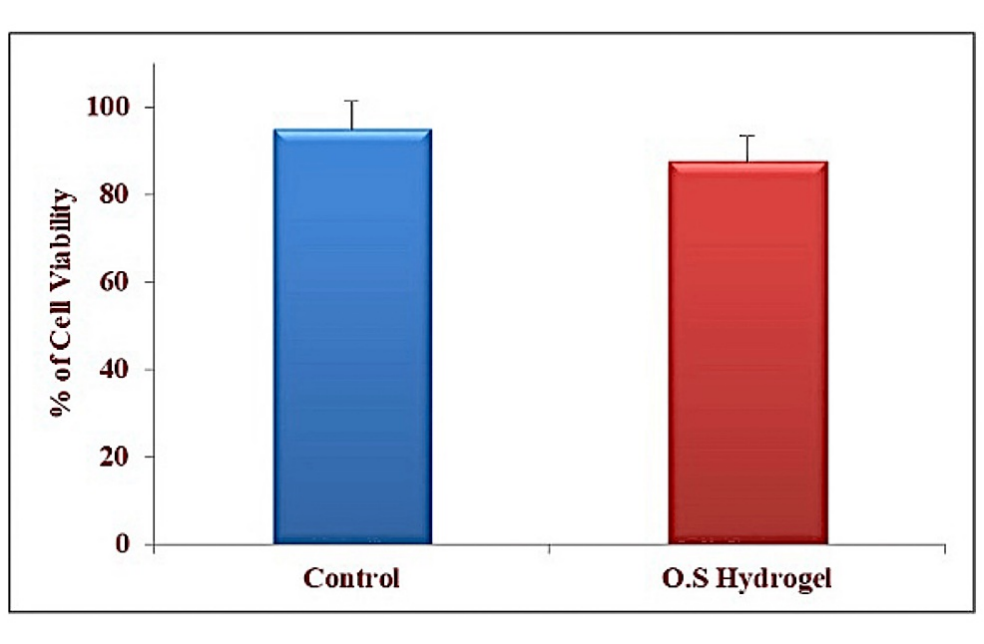
MTT assay The percentage of cell viability (MG-63 cell line) of O.S. (Ocimum sanctum) hydrogel and PBS (phosphate buffer solution) as controls was assessed.

The experimental cells (MG-63 cell line) which showed viability above 90% for control, whereas 85% for OS hydrogel was measured by a 3-[4, 5-dimethylthiazol-2-yl]-2, 5-diphenyltetrazolium bromide assay (Figure 2).

Cell morphology

**Figure 3:**
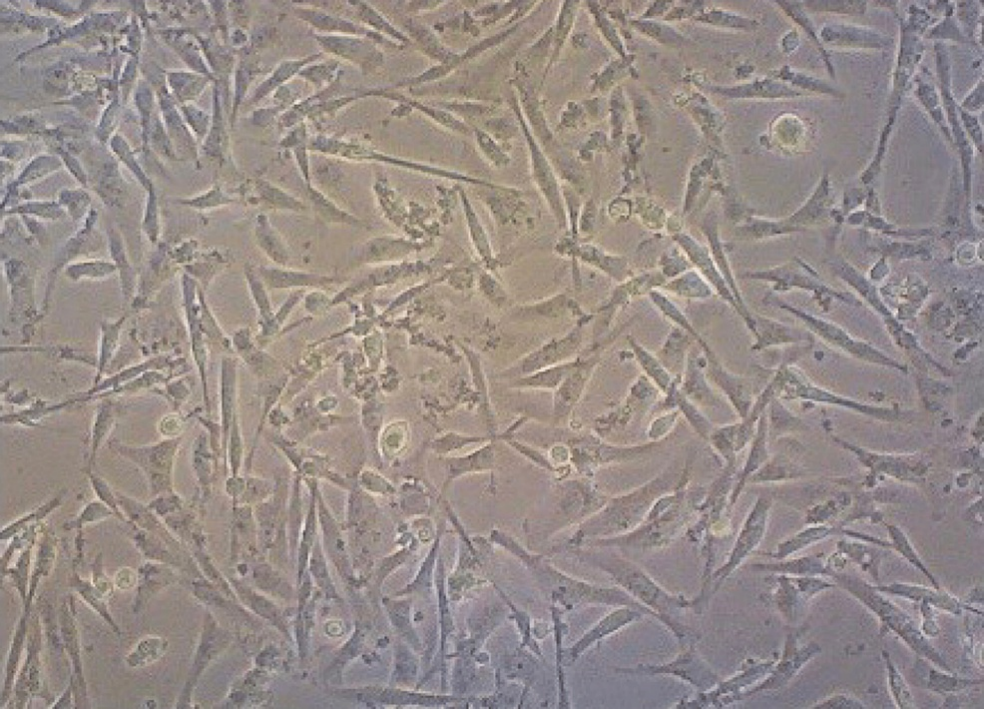
Cell morphology Uniformly shaped, spherical cells may be seen forming in a monolayer in normal MG-63 cells. Smaller, irregular nuclei and nuclear fragments are characteristics of apoptotic cells.

After the treatment with OS hydrogel at a concentration of 25 g/mL, morphological alterations that might indicate the induction of apoptosis were seen. In Figure 3, sample results are displayed. Only uniformly shaped, spherical cells may be seen forming in a monolayer in normal MG-63 cells. Smaller irregular nuclei, nuclear fragments, and red-stained cytoplasm that are characteristics of apoptotic cells are less common, which indicates less toxicity of the hydrogel (Figure 3).

Cytotoxicity test

OS gel's cytotoxicity was evaluated using the brine shrimp lethality assay. After the hatching of eggs, nauplii were examined for the prepared extract. The assay demonstrated that nauplii survived at varying OS gel concentrations. In comparison to greater concentrations of the extract, nauplii survived after 24 and 48 hours in a less concentrated extract. The nauplii survival rate was within acceptable limits (Table 1).

**Table 1:** BSLA results at baseline, 24 hours, and 48 hours at different concentrations The survival rate of Nauplii was assessed at different concentrations of OS hydrogel in microliters at baseline, 24 hours, and 48 hours.

O. sanctum extract (in µl)	Baseline	24 hrs	48 hrs
10	100%	80%	70%
20	100%	70%	60%
30	100%	70%	50%
40	100%	60%	50%
50	100%	60%	40%

## Discussion

Disrupting the biofilm and eliminating the etiological causes are the first steps in treating periodontal disease. Antimicrobial therapy is used to reduce the number of microorganisms in the local environment. Drugs given locally into the gingival crevice provide a benefit over treatments taken systemically, which have negative effects. Drugs that are locally administered should be kept in the pocket for the needed amount of time and at a concentration that is higher than the minimal inhibitory concentration for periodontal infections. For efficient medication administration, the pharmacokinetics of locally administered medicines should adhere to zero-order kinetics. Periodontal disease has been treated with drugs such as tetracycline, doxycycline, minocycline, metronidazole, and chlorhexidine, although there may be side effects. But these medications lack the ability to reduce inflammation or function as antioxidants to influence the host. As a result, efforts are being made to find a medication or natural extract with antibacterial, antioxidant, and anti-inflammatory properties [11].

O. sanctum belongs to the Lamiaceae plant family and has been recognized for its therapeutic properties since ancient times. Modern studies have also contributed to our understanding of its medicinal potential. Tulsi contains several phytoconstituents that contribute to its medicinal properties. The leaves of O. sanctum contain approximately 0.7% volatile oil, with eugenol and methyl eugenol as the major components, comprising around 71% and 20% of the oil, respectively. Also, the presence of some hydrocarbons like carvacrol and sesquiterpene increases its therapeutic properties. Additionally, O. sanctum leaves and stem extract contain various phenolic compounds like cirsilineol, cirsimaritin, isothymusin, apigenin, and rosameric acid, which act as antioxidants [12]. Research has suggested that Tulsi exhibits antibacterial, antioxidant, antifungal, and anti-inflammatory properties, primarily due to the presence of essential oils in the herb [13]. These essential oils are thought to be responsible for their effectiveness against various pathogens. Moreover, Tulsi has been proposed to have an immunomodulatory effect, potentially boosting pro-inflammatory mediators, thereby enhancing the host's defense [14,15].

The study was conducted by Katsuda et al., where they used the MTT assay to evaluate the action of grape seed extract (GSE) on human gingival fibroblasts (hGFs). The study aimed at analyzing the antioxidant properties of GSE and comparing them with the water-soluble analogue of tocopherol in patients with periodontitis. In this study, the researchers aimed to determine if GSE had cytoprotective effects on hGFs [16]. In a study by Müller et al., the MTT test on L-929 cells was used to determine the effect and cytotoxicity of Kaolinite-based solutions on human periodontal cells [17]. These studies suggested that herbal extracts are effective in the treatment of periodontitis. O. sanctum extract underwent an MTT experiment in a recent publication by Luke et al., and the results showed that O. sanctum is a cytotoxic agent against an oral squamous carcinoma cell line (Ca9-22). This plant's leaves contain phytochemicals, which give it the ability to combat oral cancer. Both the aqueous and dry extracts of the plant are efficient; however, a comparison investigation shows that the aqueous extract performs better than the dry extract [18].

In this study, the cytotoxicity of OS gel was evaluated using the MTT assay, MG-63 cell lines, and the Brine Shrimp Lethality Assay. When compared to the control chlorhexidine gel, the results showed that the cells had a nearly identical survival rate. These findings are consistent with earlier research that evaluated the cytotoxicity of Ocimum sanctum and its potential use as local drug delivery for patients with periodontitis.

Limitations

The limitations of the study include the formulation of the hydrogel with controlled release of the active compound, and the determination of an appropriate dosage and concentration should be followed by robust clinical trials specifically evaluating its effectiveness as a hydrogel for periodontitis.

## Conclusions

Ocimum sanctum has been traditionally revered for its potential medicinal properties, including its antioxidant, anti-inflammatory, and adaptogenic effects. These aspects need to be balanced with observed cytotoxicity to form a comprehensive understanding of its overall impact on periodontitis. From all the cytotoxicity assays performed in this study, it can be concluded that OS hydrogel shows low toxicity as compared to chlorhexidine gel and can be used as a local drug delivery agent for periodontitis. Although further research is needed to unravel the underlying mechanisms, assess the relevance of these findings in clinical applications, and provide a perspective on its potential benefits and risks.
